# Annotations

**Published:** 1893-10-14

**Authors:** 


### Annotations.

### Cremation at the Church Congress.

Mr. Seymour Haden, F.R.C.S., expressed his surprise that the apologists for cremation should have been given a hearing, "of all places in the world, at a Congress of the Church of England." We, for our part, are inclined to hold that, "of all places in the world, a Congress of the Church of England" is the very place in which cremation may be most fittingly discussed. It is thus that, with the amiablest of feelings towards each other, "doctors" feel constrained to "differ," and to confess their differences. Dr. Bostock Hill, of Birmingham, championed the cremationists at the Congress, and his contention, put into a few words, is that cremation accomplishes quickly what burial effects slowly, and does it better and with greater safety to the public health. Mr. Seymour Haden, as of old, championed the burialists; and his contention is, also as of old, that burial accomplishes slowly what cremation effects quickly, but does it very much better and with very much more safety and advantage to the public health. "Who shall decide when two doctors disagree?" Why, a third doctor to be sure! And the part of Solomon in this dispute was played by Dr. Vivian Poore. Dr. Poore is for freedom—for the use of burial where burial will manifestly serve the purpose, and for cremation where cremation would obviously be best. Inasmuch as this is the very position we have always taken up in this journal, we shall, of course, give our vote in favour of Dr. Poore. And really, when one comes to think of it, is not this the natural end of the controversy? We have had twenty years of Mr. Seymour Haden, Sir Henry Thompson, and other contentious scientists; they have every one said all they have to say, nothing new by any chance comes either from attack or defence. Let common sense, in the person of Dr. Vivian Poore, cry "peace"; and let both burial and cremation have their fitting places in the sanitary decalogue of the country.

### Cancer Researches.

The argument now being illustrated and enforced in the *Journal of Pathology* on the parasitic causation of cancer is admittedly of the first order of importance, though up to the present time no facts of an absolutely convincing character have been adduced by the protagonist M. Armand Ruffer. But though no definitely convincing facts are yet forthcoming, those who sympathise with arduous research excellently conducted will give M. Ruffer their unqualified admiration and gratitude. That something has been discovered in cancerous growths is beyond a doubt. That that something in appearance and behaviour, and more especially in behaviour, is very like a parasitic protozoon no careful student will deny. Its uniformity of size, of shape, and of distribution, and its apparent mode of reproduction, with other like circumstances, all point to an organism of the protozoal, and therefore also of the parasitic order. Only the second section of M. Ruffer's communication is now published, and we await succeeding communications with intense interest. In the meantime a research on this subject has been made and is published by Dr. Lindsay Steven and Dr. John Brown. There are certain things to be noted in these two practically concurrent researches which are very instructive. M. Armand Ruffer, for example, is positive; Dr. Lindsay Steven and Dr. Brown are cautious. Both the papers are illustrated by numerous exceedingly beautiful and careful drawings; but it is worthy of note that M. Ruffer's drawings bring out much more precisely and definitely the apparent facts which support the parasitic hypothesis than do those of Dr. Lindsay Steven and Dr. Brown. Is it that the microscopical appearances are actually much more clear and distinct to M. Ruffer than to the Glasgow doctors, or is it that a conviction of the truth of his own hypothesis lends exalted powers to his microscope? In any case a comparison of the two sets of drawings is interesting, and considering that both observers have been examining similar objects, it is perhaps a little amusing, of course in a highly decorous and scientific kind of way. Dr. Ruffer, not having completed his communication, does not summarise his conclusions. Dr. Lindsay Steven and Dr. Brown express their cautious opinions, as they are formed on the facts which they have observed. They say: "In conclusion, we have to say that we can go no further in the expression of an opinion as to the nature of the inclusion bodies (the supposed parasites) than to state that they appear to us to be organised elements." The two papers are both models of careful statement; and we venture to express the hope that Dr. Lindsay Steven and Dr. Brown, as well as Dr. Ruffer, will prosecute further researches on this profoundly important subject.

### Simple Methods in Surgery.

Medicine, including surgery, is a tremendously learned profession in these times; but allmen will admit that when we come to the practical part of it our methods cannot possibly be too simple, provided always that they are perfectly efficient. The *Practitioner* has an excellent article in its August number on the "Treatment of Fractures by Simple Methods," by Professor John Chiene, of Edinburgh University. Professor Chiene, in a few words of preface, tells his brother practitioners one or two elementary truths of much everyday worth. Says he: "A specially prepared splint lying in a surgeon's press, ready to spring out on the first patient who may present himself, always reminds me of a ready-made pair of trousers or coat lying nicely folded, ready to be applied to the first trouser-wanting or coat-wanting person who may present himself. This is not as it should be." Decidedly it is not. No one doubts that certain classical splints of great repute in hospitals are very valuable instruments; and it is equally true that one and the same splint may be adapted to a great many different persons by judicious manipulation. But when all is said, it still remains the fact that three-fourths of the fractures and serious injuries with which surgeons are called upon to deal lie outside the radius of immediate hospital aid. It is therefore imperative that every general practitioner should know how to promptly construct efficient splints and other instruments out of the most elementary materials. Nothing could well be more elementary than the materials which the great Syme often used to carry into his class room as an all-sufficient armamentarium for all common "solutions of bony continuity." "Syme," says Professor Chiene, "entered the clinical theatre with a bandage, a long splint and a sheet, and a Gooch splint, and proceeded to demonstrate the treatment of fractures by this simple means. Simplicity was his principle; special splints and special apparatus were put aside as useless and inexpedient." It is obvious that in a mere annotation we cannot demonstrate the application of these principles of simplicity to any considerable variety of cases. We are convinced, however, that many practioners live more or less in a surgical fog, even with regard to the elementary management of fractures. In less than a dozen pages of the *Practitioner* Professor Chiene enunciates with absolute clearness certain simple methods by which almost every ordinary fracture of bone may be successfully treated even by the surgical tyro.

